# Depth-of-Focus Correction in Single-Molecule Data Allows Analysis of 3D Diffusion of the Glucocorticoid Receptor in the Nucleus

**DOI:** 10.1371/journal.pone.0141080

**Published:** 2015-11-10

**Authors:** Rolf Harkes, Veer I. P. Keizer, Marcel J. M. Schaaf, Thomas Schmidt

**Affiliations:** 1 Physics of Life Processes, Huygens-Kamerlingh Onnes Laboratory, Leiden University, Leiden, The Netherlands; 2 Institute of Biology Leiden (IBL), Leiden University, Leiden, The Netherlands; University of Ulm, GERMANY

## Abstract

Single-molecule imaging of proteins in a 2D environment like membranes has been frequently used to extract diffusive properties of multiple fractions of receptors. In a 3D environment the apparent fractions however change with observation time due to the movements of molecules out of the depth-of-field of the microscope. Here we developed a mathematical framework that allowed us to correct for the change in fraction size due to the limited detection volume in 3D single-molecule imaging. We applied our findings on the mobility of activated glucocorticoid receptors in the cell nucleus, and found a freely diffusing fraction of 0.49±0.02. Our analysis further showed that interchange between this mobile fraction and an immobile fraction does not occur on time scales shorter than 150 ms.

## Introduction

Since the initial camera-based observation of the diffusion of individual molecules in artificial membranes [1], single-molecule imaging technology has yielded a plethora of novel insights into the behavior of proteins and other membrane constituents *in vitro* [2–4], i*n cellulo* [5–11] and *in vivo* [12]. Single-molecule microscopy has been of great importance to quantify the diffusive properties of membrane constituents. Diffusive properties consequently report faithfully about the local structural properties of the membrane, the activation state of signaling pathways [13], transport of membrane components [14], or cellular regulation processes [15,16]. For a homogeneous system in equilibrium, one would predict that the ensemble-averaged mobility is hence governed by multiple populations, each reflecting a distinct molecular state of its components. Indeed, experimental verifications of this prediction have ubiquitously been found. Whether particle-averaged mean-squared displacement analysis [17], molecular step-width distributions [18] or molecular squared-displacement distributions [19] were analyzed, multiple populations have always been found in the analysis of receptor mobility in cells.

Given that single-molecule imaging permits to follow processes in time, there have been many attempts to find transitions between states i.e. transitions in diffusive behavior. Those should show up as change in the fraction size of different mobility when changing the time of observation. Using gold [14] or quantum-dot labeling [20] of individual components, or by labeling larger structure like liposomes [21] long time scales could be covered and switching behavior has been observed.

**Fig 1:**
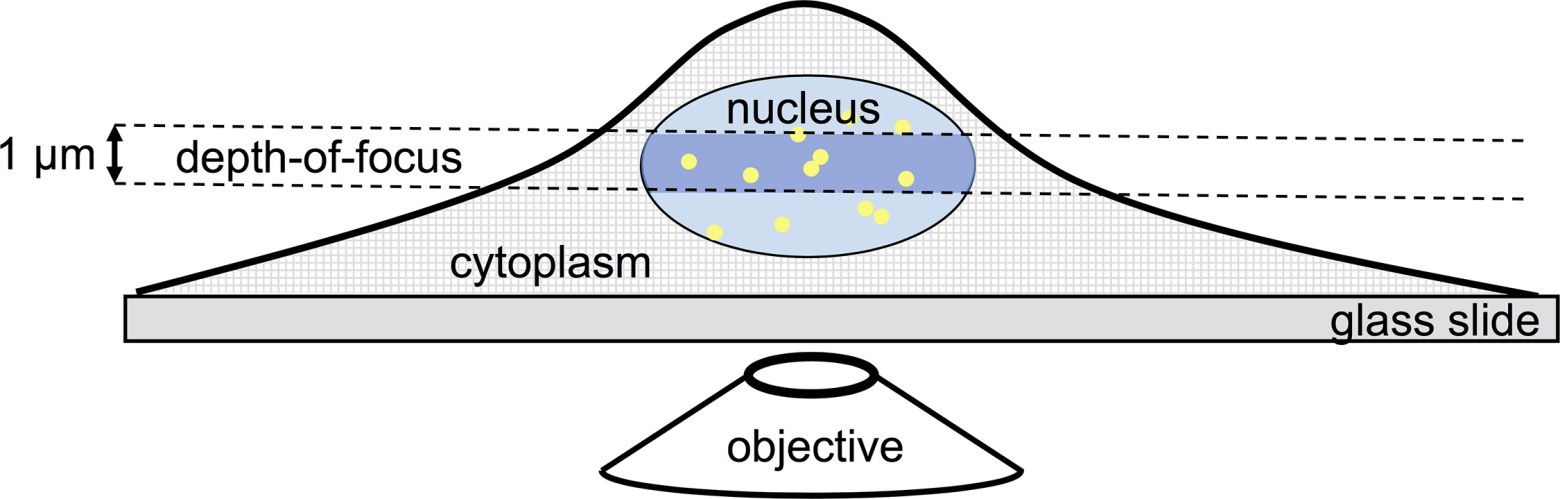
Imaging of diffusing fluorophores inside the nucleus. Since the depth of focus (DOF = 750 nm) is shallow, molecules can diffuse in and out of the observation volume. This will deplete the relative contribution of the fast diffusing fraction to the analysis.

Spurred by the success of single-molecule imaging in membrane biology and biophysics, in recent years the technology has been further developed to permit single-molecule observations of proteins in the 3D environment inside live eukaryotic cells [18,22,23]. In those experiments individual proteins were imaged over time, their position analyzed in 3D to sub-wavelength accuracy [24], and subsequently the mobility analyzed by step-length analysis. Similar to the membrane constituents, mobility of cytosolic proteins appeared inhomogeneous and fractions of different mobility were identified. Various research groups [18,22,23] realized that, unlike when imaging on the 2D membrane surface, the apparent fraction size of the various components depends on observation time. This is due to movements of molecules out of the depth-of-field of the observation volume: fast molecules will disappear faster compared to slow molecules (Fig 1). Given typical values for the depth-of-field of 1 μm for both wide-field [18,23] or selective-plane [22] illumination and typical diffusion constants of cytosolic proteins of 10 μm^2^/s, the residency time of a molecule within the observation volumes reduces to <50 ms. Hence, in those earlier reports fraction sizes for short time-lags of 6.5 ms and 20 ms, respectively, were reported to avoid any 3D artifact [18,22,23].

Here we present a mathematical framework that can correct for the change in fraction size due to the limited detection volume in 3D single-molecule imaging. We applied our findings to data on the mobility of activated glucocorticoid receptors (GR) in the nucleus of monkey kidney (COS-1) cells. Our analysis showed that fraction sizes remain constant in the time-lag range from 6.5 ms to 150 ms, thus showing that switching between fractions occurs on longer time scales.

## Methods

### Cell culture

To measure the diffusive properties of GR-eYFP COS-1 cells (acquired from ATCC) were cultured on coverslip glasses and transfected using X-tremeGENE (Roche, 500 ng DNA / 10 cm^2^) according to the manufacturers protocol. Three to six hours prior to measurement 1 μM dexamethasone (final concentration, Sigma-Aldrich) was added to the cells. Measurements were carried out at 37°C.

### Single-Molecule Imaging

Imaging of individual GR-eYFP was performed as described earlier [23]. In brief 1200, frames per cell were taken on an inverted wide-field fluorescence microscope (Axiovert 100TV) using a 100x/1.4NA oil-immersion objective (Zeiss). A region of interest of 50x50 pixels was selected (pixel size of 202 nm in the image plane). Cells were illuminated with 514 nm by a DPSL laser at an intensity of 2 kW/cm^2^ (Coherent Sapphire). The exposure time was kept constant at 3 ms and the time lag between two images varied between 6.25 and 75 ms by means of an acusto-optical tunable filter (AA optoelectronics). 45 cells were measured with 6.25 ms time lag between frames. 20 cells were measured with 25 ms lag time between frames. 16 cells were measured with 50 ms between frames. 16 cells were measured with 75 ms between frames.

The fluorescence signal from individual eYFP molecules was captured on an emCCD (Princeton Instruments, Trenton, NJ) using a combination of filters (DCLP530, HQ570/80 (Chroma Technology, Brattleboro, VT) and OG530-3 (Schott, Mainz, Germany). In order to obtain short acquisition times between frames of 6.25 ms the camera was run in kinetics-mode that permitted to capture 8 consecutive frames on the camera chip before being digitized. Subsequently, signals were fitted with a 2 dimensional Gaussian using a custom algorithm in Matlab [25]. The position of the molecules was obtained from the fitting parameters to an average accuracy of 34±9 nm. The 2D distance between localizations could therefore be obtained with an accuracy of 68 nm.

### Particle image correlation spectroscopy (PICS) analysis

At high densities a tracking algorithm mixes trajectories. The previously described method of particle image-correlation spectroscopy (PICS) circumvents this problem and is often used to analyze membrane diffusion [26].

In PICS the cumulative distribution function (cdf) of squared distances between frames separated at a given time-lag is calculated from the position data. The drop of the cdf at short distances reflects the mobility of molecules [26]. For a mobility characterized by diffusion the drop follows an exponential [19]. As has been reported earlier by us [27], the drop is faithfully described by a bi-exponential, which reflects the bi-modal behavior of the receptor: a freely diffusing receptor and an immobile, bound receptor.

For each measurement multiple time-lags are obtained by correlating not only subsequent frames but also further frames. However, due to photo bleaching the gap between frames cannot be increased indefinitely. Hence, the 6.25 ms dataset was analysed up to 5 steps (6.25–31.25 ms), the 25 ms dataset was analysed up to 4 steps (25–100 ms), the 50 and 75 ms datasets were analysed up to 2 steps (50–150 ms).

### Depth of field calibration

The depth of field (DOF) is defined by the axial offset of a point-object from the focal plane at which the width is increased by a factor √2 [28]

$$\sigma (z)\;=\sigma_{0}\sqrt{1+{\left(\frac{z}{DOF}\right)}^{2}}$$ (1)

Eq (1) shows how the width, σ, of the PSF changes with the axial distance z from the focal plane. σ_0_ is the width at the focal plane. Combining Eq (1) with the expression for the width at focus one obtains an equation for DOF, which only includes σ_0_ and the wavelength of light, λ [28]:

$$DOF\;=2\;\frac{\pi *\sigma_{0}^{2}}{\lambda }$$ (2)

**Fig 2:**
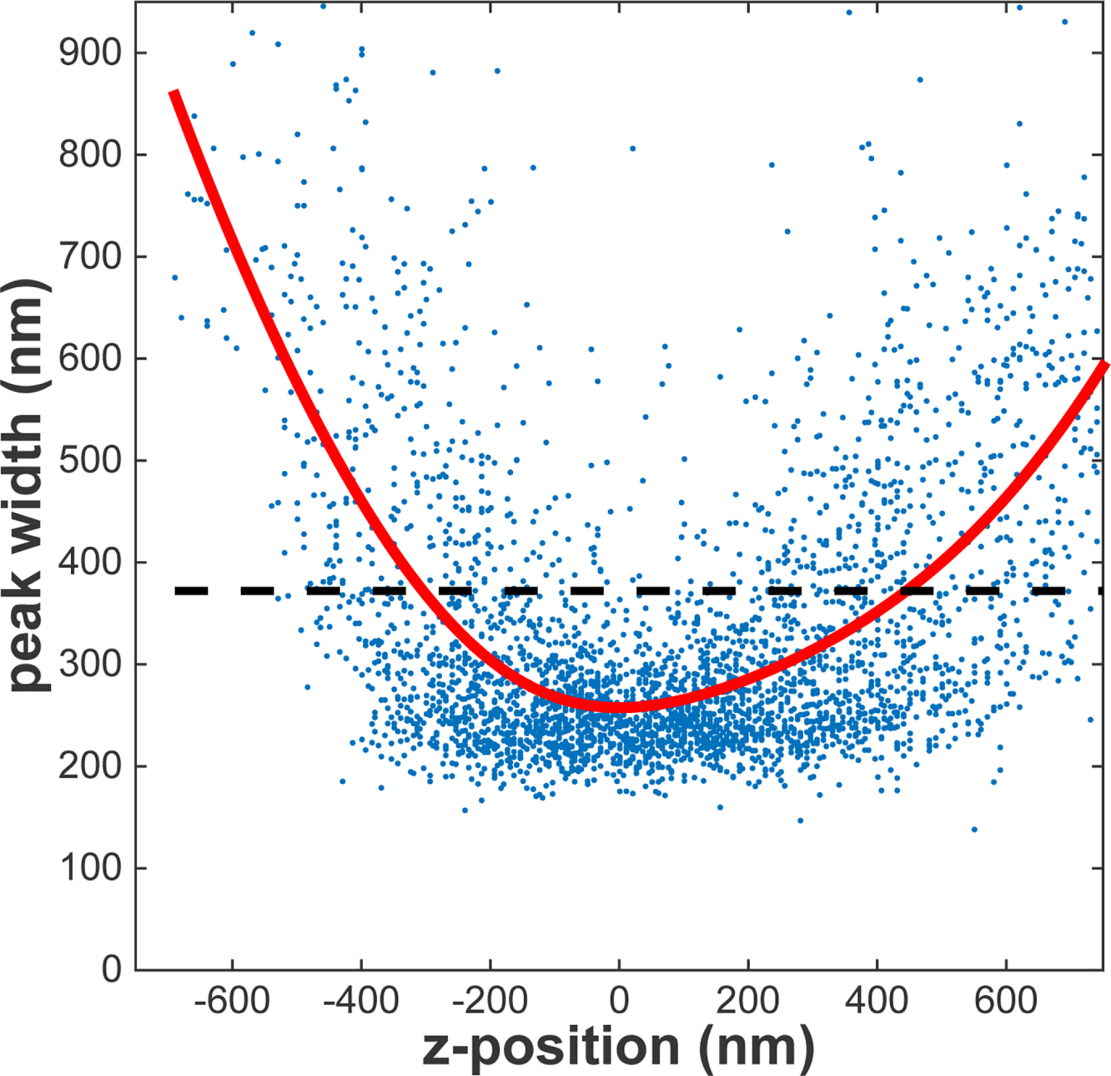
Calibration of the depth of field (DOF). eYFP was coated on a glass slide and the objective was moved by a piezo scanner (S1 Fig Data). The resulting peak-widths were fitted as previously described [25]. The data were subsequently fit to Eq (1) yielding the signal width at focus, *σ*_0_ = 263 nm and the DOF = 750 nm. All data characterized by a width larger than √2 × 263 nm = 372 nm (dashed line) were discarded from further analysis.

To experimentally obtain the DOF, eYFP molecules were coated on a glass slide. The sample was imaged for different axial positions of the objective by means of a piezo-actuator (PiFoc, PI). The fluorescent signal of single eYFP molecules was subsequently fitted [1]. From the fit the peak-width was obtained. The relation between axial position and peak-width was subsequently fitted as given by Eq (1) [25]. From this experiment the width at focus of σ_0_ = 263 nm and the DOF = 750 nm, as defined by the axial position at which the width increases by √2, was determined (Fig 2). The experimentally determined DOF is in agreement with that predicted from Eq (2) of 790 nm, given the experimental value for σ_0_ and the emission wavelength of eYFP (550 nm).

In all further analysis localizations originating from fluorescent signal of width larger than √2 × 263 nm = 372 nm were discarded.

## Results

### Analytical solution for correction of fraction size in 3D diffusion with limited detection volume

Since the width of the point-spread-function (PSF) increases with increasing distance to the focal plane, the signal from an out-of-focus object will be spread out over a larger region of the detector and the signal to noise ratio will decrease concomitantly. Therefore the detectability of a molecule is limited to a small distance from the focus defining the depth of field (DOF). The DOF was measured to be 750 nm (Fig 2). With respect to detailed mobility analysis that includes various fractions, the limited DOF will result in a bias towards the slowest fraction. Fast diffusing molecules will have a higher chance of diffusing out of the DOF than slow diffusing molecules. Therefore they will have a smaller contribution to the cumulative distance distribution.

In what follows we derive an analytical solution for a system that consist of two fractions of diffusing objects characterized by diffusion constants D_1_ and D_2_, and fractions α and 1-α, respectively. The description can easily be expanded to include more fractions.

For a molecule that is localized at axial position z_0_ the probability density for its axial location z after a time t, with a diffusion constant of D is given by:

$$pdf(z,z_{0},D,t)=\frac{1}{\sqrt{4\pi Dt}}e^{-\frac{{\left(z-z_{0}\right)}^{2}}{4Dt}}$$ (3)

Hence, the probability to stay within the DOF of length L is given by:

$$\int_{0}^{L}\frac{1}{\sqrt{4*\pi *D*t}}e^{-\frac{{\left(z-z_{0}\right)}^{2}}{4*D*t}}\;dz=\frac{1}{2}\left(\mathrm{erf}\;\left(\frac{z_{0}}{\sqrt{4*D*t}}\right)\;+\mathrm{erf}\;\left(\frac{{L-z}_{0}}{\sqrt{4*D*t}}\right)\;\right)$$ (4)

with erf being the error function. Further integration over the start position z_0_ from 0 to L results in the average probability to stay within the DOF:

$$\bar{P}(L,D,t)=\text{erf}\;\left(\frac{L}{\sqrt{4*D*t}}\right)+\frac{\sqrt{4*D*t}}{L\sqrt{\pi }}\left(e^{-\frac{L^{2}}{4*D*t}}-1\right)$$ (5)

Which finally leads to:

$$\bar{P}=\text{erf}(f)+\frac{1}{f\sqrt{\pi }}\left(e^{-f^{2}}-1\right),\;f=\frac{L}{\sqrt{4*D*t}}$$ (6)

**Fig 3:**
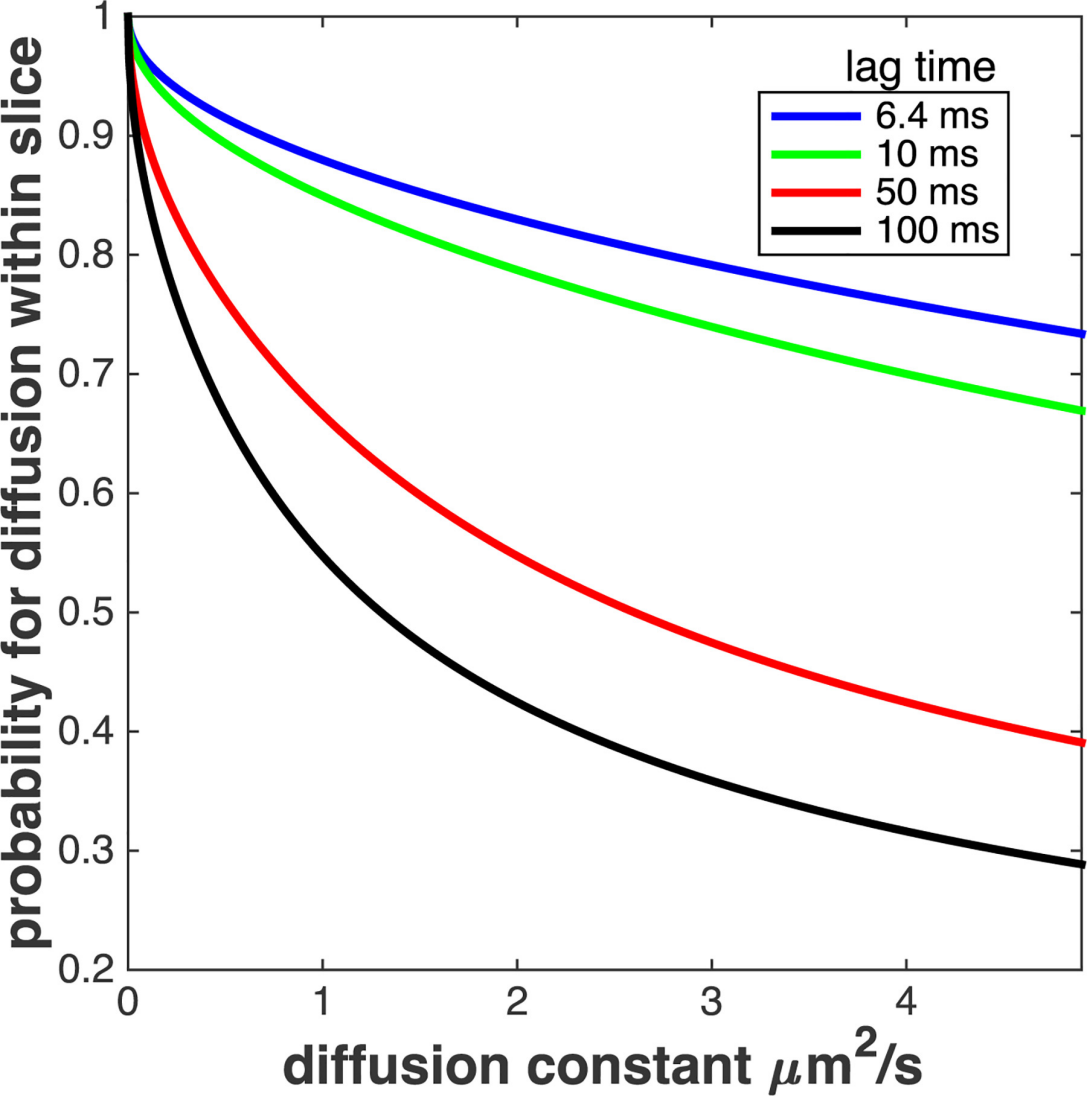
Result of Eq (6) for DOF = 750 nm and four different time lags, t (S1 Fig Data). For a diffusion constant of D = 2 μm^2^/s the probability to reside inside the DOF after t = 10 ms is 0.79, whereas for D = 0.1 mm2/s the probability is 0.97.

Eq (6) describes the probability for a molecule that started inside the DOF to still reside within DOF after time t. Fig 3 shows the functional form for a realistic DOF of 750 nm and imaging time from 6.4 to 100 ms. The probability strongly depends on D, reducing even for short imaging times of 6.4 ms from P(0.1μm^2^/s) = 0.96 to P(2μm^2^/s) = 0.83, the range of diffusion constants typically reported. Following Eq (6) this effect becomes even more pronounced for longer imaging times.

In what follows we describe how Eq (6) is used to calculate the actual fraction size from imaging data in the case of multi-modal inhomogeneous diffusion data. By PICS analysis D_i_, and the apparent fraction size, α_i_, are obtained. Together with Eq (6) the real fraction sizes, β_i_, are obtained:

$$\beta_{i}\;=\frac{x_{i}}{\sum_{i}x_{i}},\;x_{i}=\alpha_{i}P_{i}$$ (7)

As required, both real and apparent fraction sizes are normalized quantities Σα_i_ = Σβ_i_ = 1.

For a two-component system Eq (7) simplifies to:

$$\beta =\frac{\alpha P_{2}}{(1-\alpha )P_{1}+\alpha P_{2}}$$ (8)

where β refers to the faction with diffusion constant D_2_, and 1-β the fraction with diffusion constant D_1_.

### Validation of the correction by simulations

To prove the correction method Monte-Carlo simulations were performed. 3000 molecules were split in two equal fractions, β = 1-β = 0.5. The fractions were characterized by diffusion constants of D_1_ = 2 pix^2^/frame and D_2_ = 0.05 pix^2^/frame, respectively. Those values were chosen based on values typically found for diffusion of proteins in mammalian cells, and in particular are equivalent to the values for the bound and unbound fraction of the glucocorticoid receptor in the nucleus (2 and 0.5 μm^2^/s) reported earlier [23]. The objects used in the simulation were free to diffuse for 100 frames in a cube of 100×100×100 pixels. Circular boundary conditions were applied. In order to set a DOF, only molecules within a slice of 5 pixel width (i.e. 1 μm) at the centre of the cube were considered.

**Fig 4:**
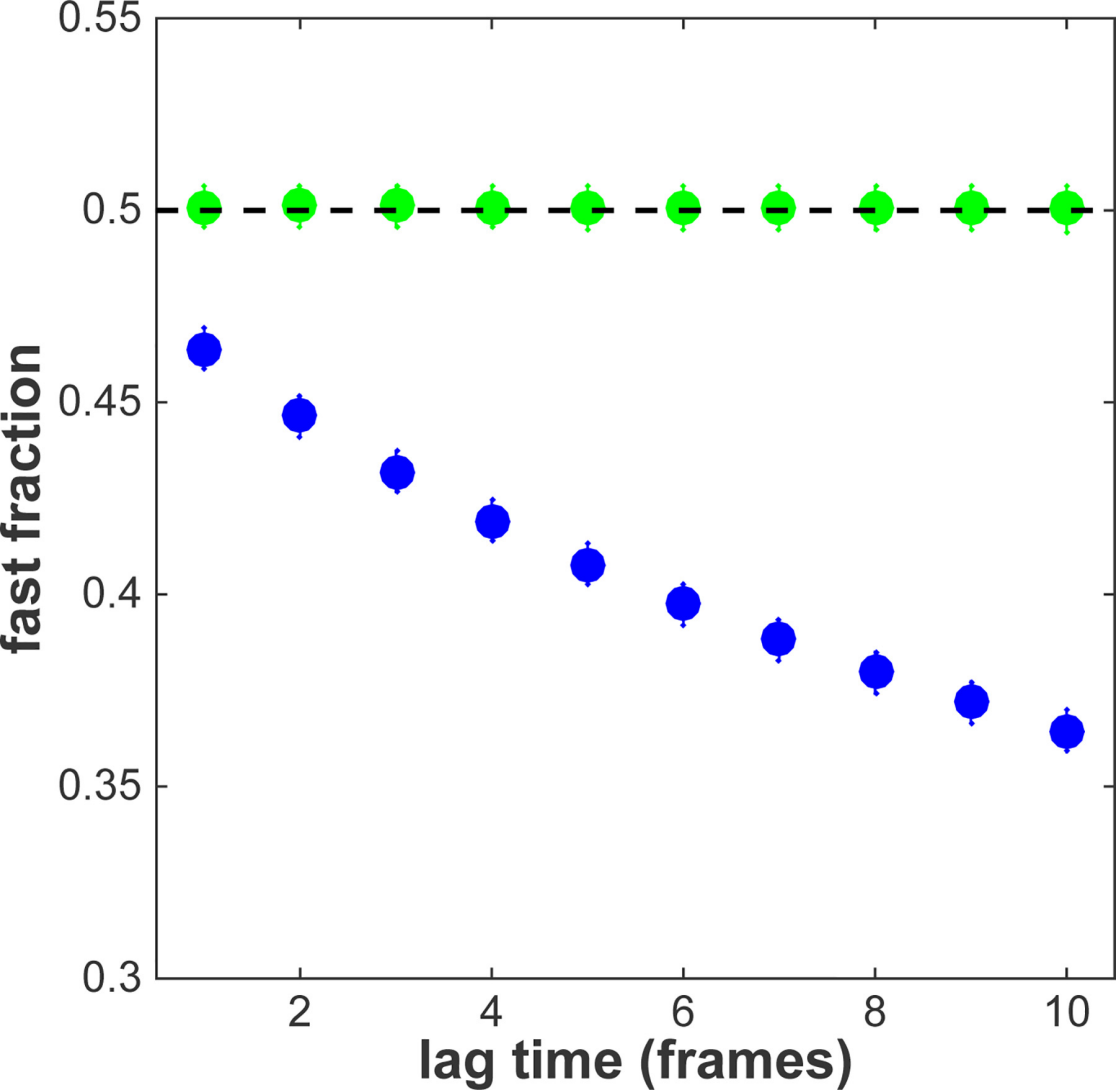
Simulation result that shows depletion of the fast fraction for increasing time lags (S1 Fig Data). The time lag is given by the number of frames between detections. In blue the uncorrected result, in green the result after correction with Eq (8).

Particle image correlation spectroscopy (PICS) utilizes the distance distribution of the diffusing particles. To analyse the simulation the distances originating from fast diffusing objects were summed and divided by the total number of distances found. The observed fractions were extracted for time lags of 1 to 10 frames. Fig 4 shows the result of this analysis (blue data). The apparent fraction size of the fast fraction decreased from 0.46±0.01 at the first time lag to 0.36± 0.01 for the 10^th^ time lag. Subsequently Eq (8) was used to correct the data and calculate the real fraction size. Fig 4 shows that our analysis faithfully follows the prediction and the real fraction size remains constant at β = 0.500±0.007 over the whole range of time lags (green data).

### Validation of the correction using experimental data

To further prove our correction method we applied the model for the correction of experimentally acquired life-cell data. The diffusion of the glucocorticoid receptor (GR) in live cells is a well-documented example for mobility of multiple fractions in a 3D environment. The GR is an member of the steroid receptor family [29–31]. It mediates the effects of natural as well as synthetic glucocorticoids like dexamethasone and prednisolone, which are drugs known for their anti-inflammatory activity that is beneficial to treat diseases like asthma and rheumatoid arthrosis [29]. Upon activation by glucocorticoids the receptor translocates from the cytoplasm to the nucleus. There it acts as a transcription factor. It binds to specific target sequences in the DNA to activate gene transcription.

The targeted search mechanism along DNA that activate or repress gene activation by hormone receptors like the GR has long been studied in theory and experiment [32]. The GR displays long immobilization times (2.3 s). The immobilized fraction probably reflects receptors bound to DNA in order to activate transcription. In addition, the GR is found to also have short immobilization times (0.7 s) [27]. Most likely the short immobilization times represents a search mechanism that includes non-specific DNA binding [31]. Finally, approximately half of the GR population shows fast free 3D diffusion [27].

Here we followed the wide-field single-molecule imaging strategy of Groeneweg et al. [27] to analyze the diffusion properties of activated GR and extended our analysis up to 150 ms time-lags. Obviously, our current approach does not discriminate between the short (0.7 s) and long (2.3 s) immobilization times of the receptor due to the short time scale of the experiments. Hence, only two fractions were distinguished, an immobile and a freely diffusing fraction.

**Fig 5:**
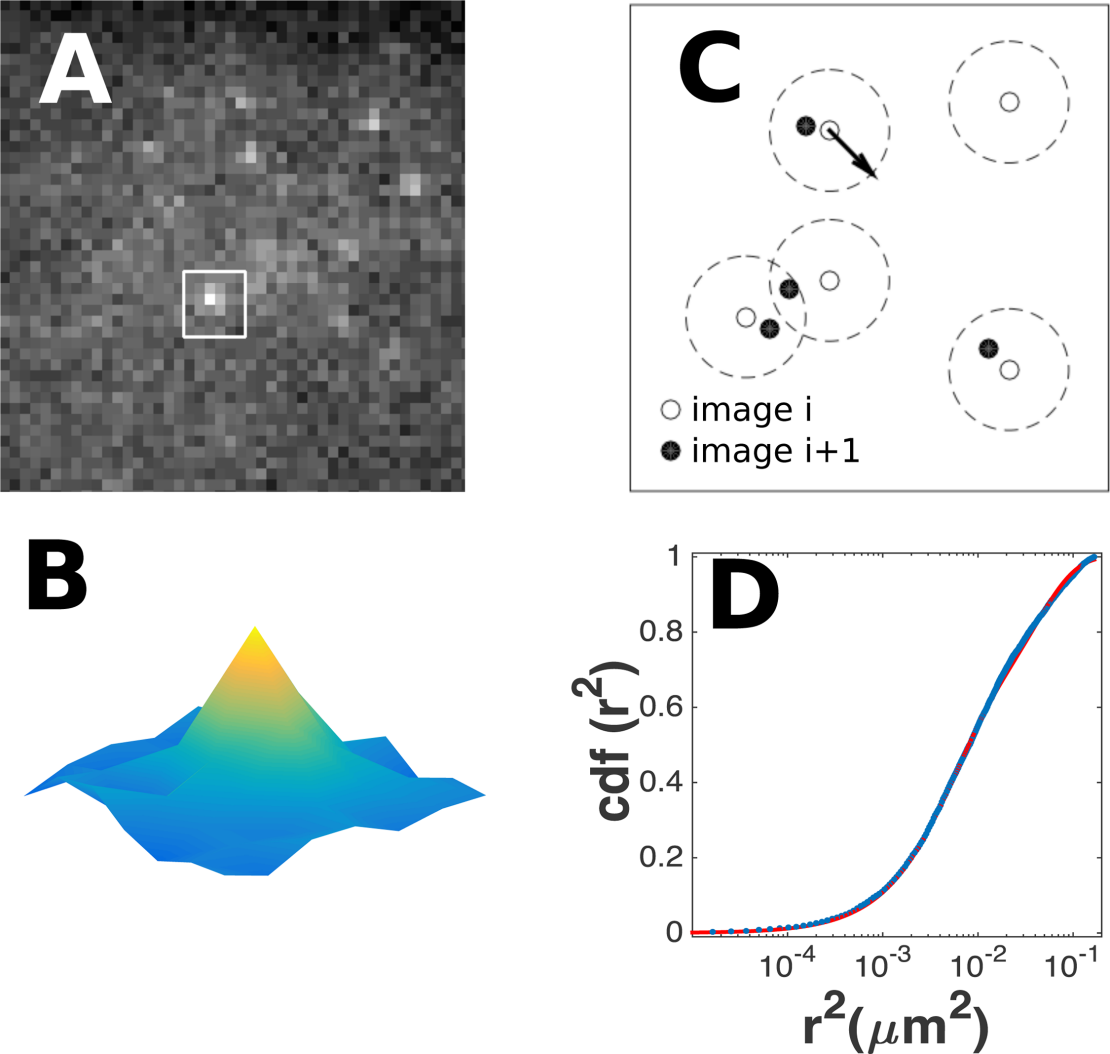
Single-molecule imaging and PICS analysis (S1 Fig Data). *A*: Signal of individual eYFP-GR molecules on an emCCD camera. *B*: The signal of an individual molecule is fitted to a Gaussian yielding the position, the width and the strength of the signal. *C*: Distance calculation between molecules in subsequent frames. *D*: Cumulative distribution function (cdf) of distances of molecules in subsequent frames correlated by diffusion.

Below briefly stated are the steps taken to obtain data on GR mobility, which are also depicted in Fig 5. COS-1 cells were transfected with a plasmid encoding a YFP-labeled version of the GR. The functionality of the plasmid has been tested previously [27]. Cells were stimulated with 1 μM of dexamethasone which leads to efficient activation and translocation of the GR to the nucleus. Subsequently, individual YFP-GRs were followed using single-molecule microscopy in which a mid-slice of 750 nm thickness of the nucleus was imaged (Fig 5A; see the DOF subsection in M&M). Individual GRs appeared as diffraction-limited images of a signal of 203±90 counts when illuminated for 3 ms at an intensity of 2 kW/cm^2^ of 514 nm light (Fig 5B). This signal allowed us to track the receptors at a lateral accuracy of 34 nm. The axial position was lost as the camera imaged the 2D projection of the 3D slice in the nucleus.

Subsequently PICS analysis was used to analyze the mobility of the GR (Fig 5C). In PICS the cumulative squared-distance distributions (cdf) in subsequent frames is calculated from position data (Fig 5D). The drop of the cdf at short squared-distances reflects the mobility of the molecules [26]. For diffusion the drop follows an exponential [19]. As has been reported by us earlier [27], the drop is faithfully described by a bi-exponential, which reflects the bi-modal behavior of the receptor: a freely diffusing receptor and a bound receptor.

PICS analysis was performed for time lags between 6.25 and 150 ms. For each time lag the diffusion constant and apparent fraction size of the two components was determined. The diffusion constants were found to be 0.67±0.1 and 0.043±0.004 μm^2^/s for the fast and immobile fraction, respectively. Our data are in excellent agreement to our earlier findings [27], and the prediction for a free diffusion process. It should be noted that the immobile fraction found in single-molecule experiments consist of two sub-fractions which can be distinguished only at time-lags beyond 1 s as accessible by fluorescence recovery after photobleaching (FRAP) experiments. In FRAP it was found that those two fractions reflect two binding modes of the receptor to DNA, are equal in size, and are characterized by immobilization times of 0.7 and 2.3 s, respectively [31].

**Fig 6:**
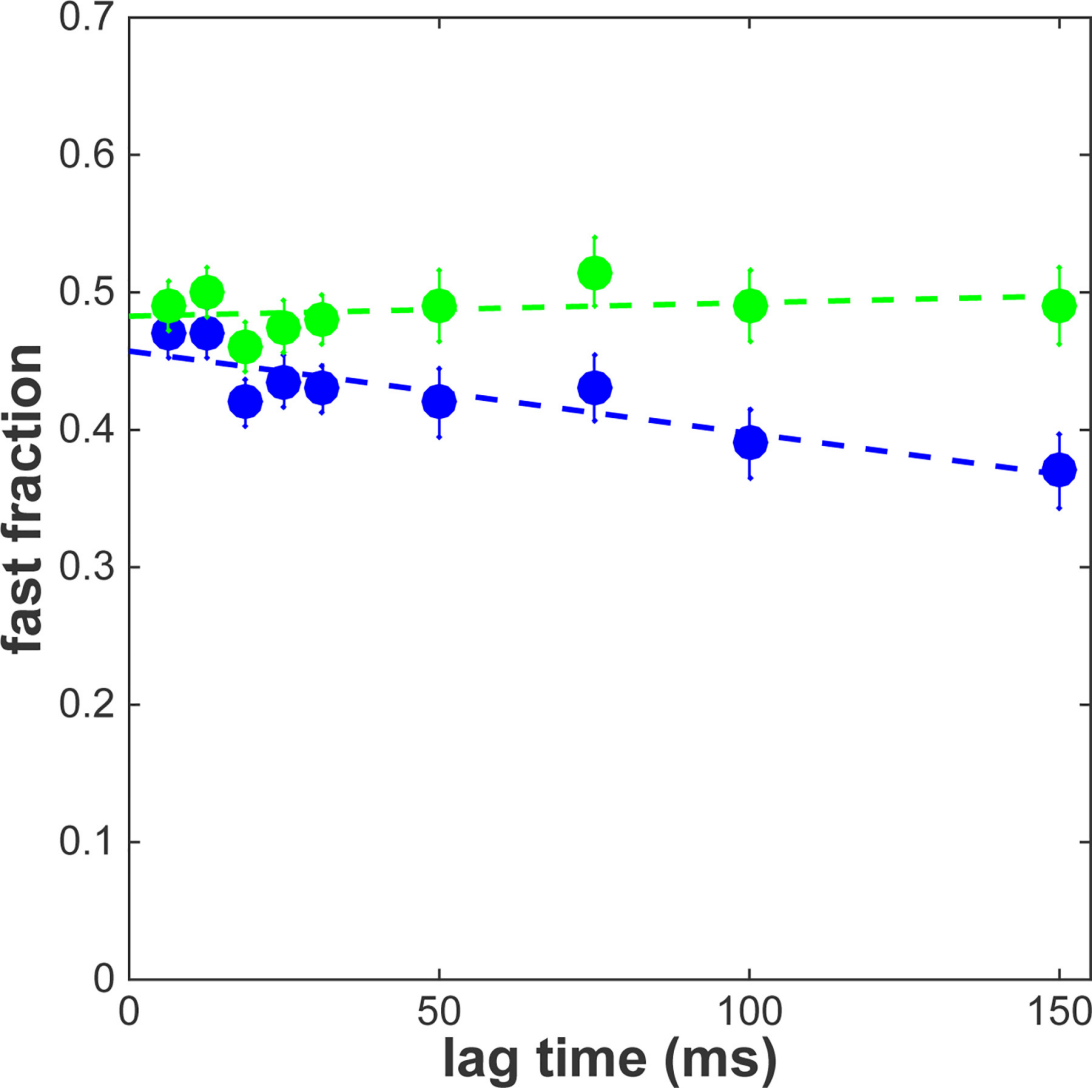
PICS analysis of glucocorticoid receptor at different time lags (S1 Fig Data). In blue the uncorrected result. A decrease of the fast fraction is observed. In green the result corrected by Eq (8) taking into account the DOF. The fast fraction stays constant for time lags at least up to 150 ms. Dashed lines are linear fits to the data. Error-bars represent the standard deviation.

As previously observed [27] the apparent fraction of the fast fraction α dropped from 0.46±0.02 at 6.5 ms to 0.37±0.02 at 150 ms (Fig 6, blue data). After correction to the real fraction size, as given by Eq (8), it is obvious that the size of the two fractions does not change in the time frame between 6.5 and 150 ms (Fig 6, green data). The real fraction size is constant and amounts to β = 0.49±0.02.

Even though the different mobility modes for various transcription factors have been repeatedly reported, it has remained challenging to address the timescales on which switching between the modes occurs [22,23,27,33–35]. The observed drop in fraction size in the uncorrected data could have been misinterpreted as an indication of switching behavior. However, since in the corrected data the fraction size does not change with increasing time lag, we conclude that switching between the two modes does not occur within the time frame of 150 ms.

## Conclusion

We showed that a depletion of fast mobile fractions is observed when multiple diffusive fractions are analysed using imaging methods that have limited axial reach. We developed a mathematical framework to correct for the experimental limitations that allowed us to calculate the real fraction sizes. Results have been validated by simulation and applied to experimental data of the activated glucocorticoid receptor in the cell nucleus. These results show that the reduction of the fast fraction with time lag, observed for the uncorrected data, is faithfully rectified by using the novel correction method. The corrected data indicate that the size of the freely diffusing fraction of dexamethasone-activated glucocorticoid receptors in the cell nucleus is 0.49±0.02. Since the corrected data show that this fraction size is constant for at least 150 ms we conclude that the receptor does not switch between this freely diffusing and an immobile (DNA-bound) state on this time scale. Thus, our theoretical framework not only allows the determination of correct fraction sizes, but provides information on potential time scale for exchange between various fractions.

## Supporting Information

S1 Fig Data All figure data are included as Matlab figure-files. The original imaging data can be obtained from the corresponding author on request.
(ZIP)
